# Capping the Pulp

**Published:** 1901-06

**Authors:** 


Capping the Pulp.—Dr. Mendel Joseph, chief of the histological laboratory of l'École dentaire de Paris, in a paper read March 6 before the Odontological Society of Paris, deplores the frequency with which practitioners devitalize the pulp. He calls attention to the remote ills that may in certain cases be attributed to devitalized teeth. He divides cases of caries involving the pulp, suitable for capping, into those (1) where there is a thin layer of dentine protecting the pulp, (2) where the pulp is laid bare by the operator, and (3) where the pulp is exposed by the progress of decay without symptoms of inflammation.

"The sterilization of the dentine in the immediate neighborhood of the pulp calls for great precaution. Antiseptics used must be neither irritant nor caustic to the pulp. Its normal functions already disturbed by the progress of decay, by the presence of bacteria, and the chemical changes to which they give rise, the pulp will not endure a new irritation. Our efforts should be to bring it back to a normal state, to quiet its sensitiveness. How, indeed, can one expect so delicate an organ to exist for a long time in the presence of the repeated or permanent irritation that it is often sought to obtain in order to favor the formation of secondary dentine? One should not forget that this formation partakes of the nature of a pathological process, and should be kept, if possible, within safe limits.

"It is to ill-considered efforts made to produce a reaction of the pulp that one must lay the blame for all the forms of degeneration of the pulp, especially the calcic form, with the formation of pulp-stones,—one of the principal causes of failure, in my opinion, in capping pulps.

"In the presence of an organ so delicate the part of the antiseptic should be to modify rather than destroy the bacteria. A substance that will kill the microbe will be apt to kill the pulp as well. To make slow progress is to make sure progress. The dressing should be renewed several times. The object of the first dressing is to modify the medium in which the bacteria are multiplying, the second to arrest their development, the third and fourth to render them utterly inert.

"My personal observations in the capping of pulps in the manner about to be described goes back eighteen months, at which time I hit upon a filling-material that was admirably adapted to make the proceeding easy and successful,—plaster of Paris. For the perfect sealing of an antiseptic dressing of a liquid or semi-liquid nature nothing can equal it; gutta-percha does not adhere to surfaces that are wet, and its insertion at such times is by no means easy, and, when it becomes necessary to change the dressing, can be removed only with the greatest difficulty. In short, it is owing to this material that I find myself able to make a successful modification of the treatment of exposed or inflamed pulps.

"My observations have led me to the conclusion that failure in pulp-capping is due to too great irritation from the dressings used, insufficient sterilization, or too much pressure on the pulp. In capping a pulp, one ordinarily excavates carefully, leaving, if possible, a layer of dentine over the pulp. He washes out the cavity with an antiseptic, applies an antiseptic dressing, and covers with cement. If there is an actual exposure, it is better to put the dressing in a little metal cap in order to avoid pressure on the pulp; this cap, by the way, is very apt to stay anywhere but upon the pulp.

"What is the ultimate result from this treatment? The infected dentine that has been left, for fear of uncovering the pulp too freely, is not made sterile by the one dressing, and as a result the micro-organisms continue to multiply, and inflammation and death of the pulp ensue.

"It is desirable, then, to succeed in completely sterilizing the cavity and to guard against any pressure on the pulp."

His method, in as few words as possible, is this: The cavity is excavated, washed, and dried as carefully as possible, and a pledget of cotton wet with chloroform placed therein while the dressing is being mixed. The dressing is made as follows:

Zinci oxidi, gr. ii;
Cocainæ hydrochlorati, gr. i;
Guaiacol, q. s. to make a creamy paste.

With this paste are incorporated some fibres of asbestos to give it a little body. The paste is put into the floor of the cavity, after removing the cotton and chloroform, without pressure, is covered with a disk of asbestos paper, and sealed with plaster of Paris. This dressing must be changed at the end of twenty-four hours. In the second dressing two grains of salol take the place of the cocaine. This dressing is left sealed, as before, with plaster of Paris, forty-eight hours; it is then replaced by a third of the same composition, which is allowed to remain four or five days, at the end of which time the final dressing of a paste of guaiacol and oxide of zinc alone, mixed as thick as possible, with the asbestos incorporated, is inserted and covered with the plaster. When the plaster has set it is covered with whatever one desires; if with gold, a layer of cement should be put over the plaster of Paris before the gold is inserted.

Dr. Joseph has treated ninety cases in this way, some of which gave evidence of more or less hyperæmia, without, thus far, any failures.—Briggs.

